# State-of-the-Art Research and New Pharmacological Perspectives on Renal Involvement in Duchenne Muscular Dystrophy: A Narrative Review

**DOI:** 10.3390/biomedicines14010230

**Published:** 2026-01-21

**Authors:** Michela De Bellis, Paola Imbrici, Roberta Lenti, Antonella Liantonio, Annamaria De Luca

**Affiliations:** Section of Pharmacology, Department of Pharmacy-Drug Sciences, University of Bari “Aldo Moro”, Via Edoardo Orabona, 4, 70125 Bari, Italy; paola.imbrici@uniba.it (P.I.); roberta.lenti@uniba.it (R.L.); antonella.liantonio@uniba.it (A.L.)

**Keywords:** Duchenne muscular dystrophy, renal dysfunction, kidney involvement, cardiorenal syndrome, dystrophin deficiency, nephrotoxicity, urinary tract abnormalities, renal biomarkers, cystatin C, multidisciplinary care

## Abstract

**Background**: Although Duchenne muscular dystrophy (DMD) is primarily characterized as a skeletal muscle-wasting disorder, the resulting pathophysiological changes extend to multiple non-muscle tissues and organ systems. Among these, renal and urinary tract dysfunctions have been reported, albeit in relatively few studies, as potential complications in DMD patients, sometimes occurring from an early age. Importantly, as life expectancy improves, the incidence of renal impairment is also expected to increase. This narrative review summarizes the available evidence on kidney involvement in DMD and discusses the associated biomarkers of renal dysfunction within the context of multisystem disease progression. **Methods**: The review draws on data from both human and animal studies and analyzes published evidence to explore kidney involvement in DMD, with a focus on clinical manifestations, biomarkers of renal dysfunction, and potential pathogenic mechanisms. **Results**: Available data indicate a close association between cardiac and renal dysfunction, particularly in patients with advanced-stage DMD. The review explores potential underlying mechanisms of renal impairment, including intrinsic dystrophin deficiency in the kidney, secondary effects of cardiovascular complications, and the nephrotoxic impact of drug therapies, highlighting renal function as an active determinant of clinical risk. **Conclusions**: While cardiac function monitoring is already a cornerstone of multidisciplinary care for this multisystem disease, systematic assessment of renal function should also be implemented, with implications for clinical management and drug safety. Moreover, the risk of drug-induced nephrotoxicity warrants attention in both clinical management and the development of novel therapeutic strategies for DMD.

## 1. Introduction

**Figure 1:**
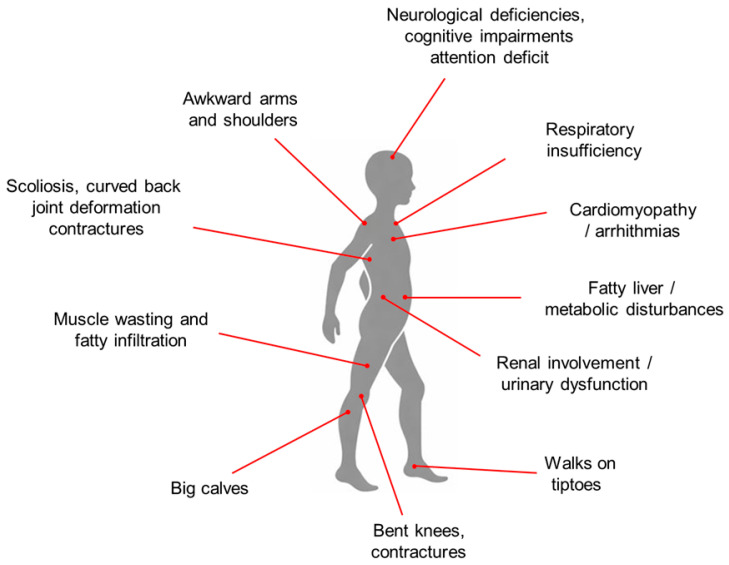
Multisystemic manifestations of Duchenne muscular dystrophy (DMD).

Duchenne muscular dystrophy (DMD) is a severe neuromuscular disorder characterized by complex disturbances of whole-body homeostasis. It is caused by mutations in the dystrophin gene (*DMD*, *300377), which result in the absence of functional dystrophin [1,2,3]. Dystrophin is a key cytoskeletal protein whose loss leads to disruption of the dystrophin–glycoprotein complex (DGC), thereby impairing force transduction and causing progressive muscle weakness and damage, accompanied by chronic inflammation and inefficient regeneration [1,2,3,4,5]. Clinically, DMD is typically diagnosed around the age of five years. Patients usually lose ambulation and become wheelchair-dependent around 12 years of age. In patients receiving early multidisciplinary interventions, assisted ventilation is usually not required until the second or third decade of life. With optimized standards of care, life expectancy can extend into the fourth decade, with mortality primarily due to cardiac and/or respiratory failure [1,2,3,4,5,6]. Although DMD is classically defined by progressive skeletal muscle degeneration, the absence of dystrophin has far-reaching consequences that affect multiple non-muscle tissues and organ systems. These include (i) progressive muscle wasting with fatty infiltration, chronic inflammation, and fibrosis; (ii) scoliosis, joint deformities, and contractures; (iii) respiratory insufficiency; (iv) late-onset cardiomyopathy; (v) neurological impairments such as cognitive dysfunction, emotional disturbances, and attention deficits; (vi) endocrine, metabolic, and mitochondrial abnormalities; (vii) gastrointestinal disturbances; (viii) fatty liver disease; and (ix) renal and urinary tract dysfunction. These systemic manifestations have been extensively documented through monographs and primary studies. In particular, the comprehensive review by Ohlendieck and Swandulla (2021) provides a broad overview of the multisystemic pathophysiology of DMD, highlighting the underrecognized involvement of kidney function [7]. The body-wide engagement underscores the need for an integrative, multi-organ approach to fully understand the complex clinical presentation of the disease, as schematically summarized in Figure 1, which frames renal involvement within the broader context of DMD as a multisystem disorder.

**Figure 2:**
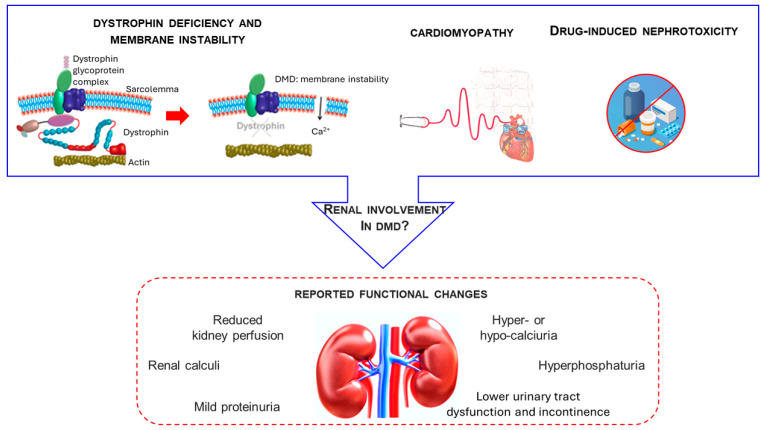
Potential determinants and renal functional changes in Duchenne muscular dystrophy (DMD).

As survival in patients with DMD continues to improve, renal dysfunction is becoming an increasingly relevant clinical concern [8]. Nevertheless, the kidney remains one of the least explored organs in the multisystemic context of DMD. This review addresses this gap in knowledge by examining renal involvement across different stages of disease progression. Specifically, we focus on potential mechanisms including primary dystrophin deficiency in the kidney, secondary effects of cardiovascular complications, and the nephrotoxic impact of long-term pharmacological treatments, as conceptualized in Figure 2, which highlights the convergence of these factors in shaping renal vulnerability in Duchenne muscular dystrophy.

### Literature Search Strategy

This narrative review is based on a non-systematic literature search conducted in PubMed and Scopus, covering publications up to early 2025. The initial search yielded over one hundred records. Articles were preliminarily screened based on title and abstract, and those considered relevant to renal involvement in Duchenne muscular dystrophy were selected for full-text evaluation. Selection was guided by thematic relevance, methodological quality, and contribution to the understanding of pathophysiological mechanisms, clinical manifestations, or biomarkers of kidney dysfunction in Duchenne muscular dystrophy, rather than by predefined formal inclusion or exclusion criteria. Additional relevant publications were identified through manual screening of the reference lists of selected articles. Both clinical and preclinical studies, as well as pertinent review articles, were considered.

## 2. Renal Involvement in Duchenne Muscular Dystrophy: Pathophysiology and Clinical Aspects

In this section, we synthesize current clinical and experimental evidence on renal involvement in Duchenne muscular dystrophy, integrating pathophysiological mechanisms, biomarkers, and pharmacological considerations.

### 2.1. Role of Dystrophin in the Kidney and Possible Consequences of Dystrophin Loss

To date, it remains uncertain whether dystrophin deficiency exacerbates renal disease in DMD. Recent evidence indicates that the biological functions of the dystrophin–glycoprotein complex (DGC) extend beyond skeletal muscle, encompassing developmental and tissue-specific roles in multiple non-muscle organs [9].

The kidney expresses the dystrophin–glycoprotein complex (DGC), which includes isoforms of dystrophin, utrophin, dystroglycan, syntrophin, and dystrobrevin [10]. Dystroglycan is expressed early by epithelial cells in the developing kidney, whereas in adult tissue its levels are markedly reduced, suggesting a predominant role in branching epithelial morphogenesis rather than in the mature organ [11]. In line with this concept, dystroglycan is increasingly recognized as a multifunctional protein that acts not only as an adhesion scaffold but also as a signaling hub in non-muscle tissues [12]. In the normal rodent kidney, full-length dystrophin (Dp427) and its shorter isoforms, Dp140 and Dp71, are expressed, although they display distinct temporal and spatial distribution patterns. The canonical Dp427 isoform is detectable in the kidney but at markedly lower levels than in skeletal or cardiac muscle, and it does not represent a major tubular isoform [11,13].

In the developing and adult brain, Dp140 expression peaks during fetal and early postnatal stages and subsequently declines [14,15]. Similarly, in the kidney, Dp140 is transiently expressed during tubulogenesis and becomes undetectable in adult tissue [11]. In contrast, Dp71 is the predominant dystrophin isoform in the adult kidney, where several alternatively spliced variants, including C-terminally truncated forms, are present at low to moderate levels [16].

With regard to protein localization, Dp71 and, transiently during development, Dp140 have been reported in renal tubular epithelial cells, particularly along the basolateral membrane, whereas Dp427 shows only limited presence in these compartments [13]. These isoforms are thought to contribute to epithelial membrane stability and to the anchoring of transporter proteins and ion channels. It is therefore plausible that the loss of specific isoforms, depending on the mutation site within the *DMD* gene, could lead to pathological alterations in tubular epithelial structure or function.

However, this mechanism does not readily account for abnormalities observed in renal corpuscles, where constitutive dystrophin expression has not been demonstrated to date [17]. In summary, whether dystrophin mutations directly contribute to kidney damage remains an open and intriguing question. On the one hand, the presence of shorter dystrophin isoforms in specialized renal endothelial cells supports a potential role for dystrophin in glomerular homeostasis [18,19,20]. On the other hand, no overt histopathological abnormalities were observed even in dystrophin–utrophin double-knockout mice [21], suggesting that the modest renal functional changes occasionally reported in mdx mice may result from secondary factors rather than direct dystrophin deficiency [22]. From a clinical perspective, this interpretation is consistent with the current view of Duchenne muscular dystrophy as a multisystem disorder, in which endocrine, metabolic, and systemic alterations may increase renal susceptibility to secondary injury rather than cause primary dystrophin-dependent structural kidney damage [23].

### 2.2. Renal Impairment in Advanced Stage DMD

Within the multisystem framework of Duchenne muscular dystrophy, the interplay between cardiac dysfunction and renal impairment can be conceptually framed as a disease-specific cardiorenal syndrome, in which progressive cardiomyopathy contributes to renal vulnerability through hemodynamic, neurohormonal, and inflammatory mechanisms [24,25]. Accordingly, renal involvement in Duchenne muscular dystrophy should be interpreted not as an isolated complication, but as part of a broader organ network characterized by dynamic crosstalk among skeletal muscle, heart, and kidney. In a limited number of studies, kidney dysfunction has been increasingly recognized in patients with advanced-stage Duchenne muscular dystrophy, although available data remain limited. In small case series of non-ambulatory DMD patients, acute renal failure has been reported, suggesting that renal complications may contribute to morbidity and potentially to mortality in advanced-stage DMD, although robust population-level data are lacking [8]. Non-ambulatory DMD patients are particularly at risk of reduced renal perfusion due to disease-related cardiomyopathy and chronic hypotension, which may predispose them to pre-renal failure [8]. At present, no specific recommendations for managing renal complications in DMD are available in the published literature or in international standards of care [2,3,4]. Likewise, histopathological evidence is lacking, as renal biopsies are rarely performed in DMD. To date, only a single report has documented an autosomal-dominant familial form of focal segmental glomerulosclerosis occurring in a patient with Duchenne muscular dystrophy [26].

**Figure 3:**
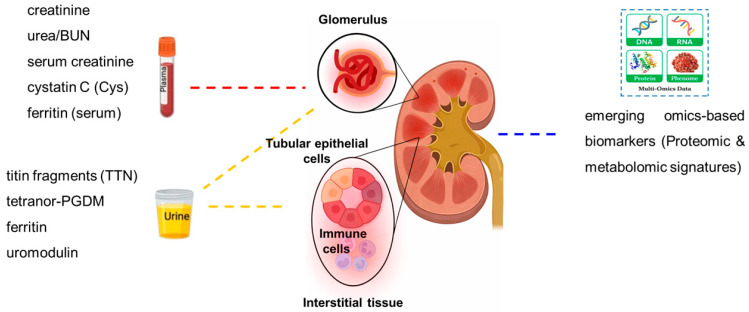
Biomarkers associated with renal involvement in Duchenne muscular dystrophy (DMD).

As illustrated in Figure 3, the assessment of renal and urinary tract involvement in DMD relies on a combination of plasma and urinary biomarkers, reflecting both the limitations of creatinine-based measures and the potential value of alternative markers in detecting subclinical renal dysfunction. In adults with DMD, serum creatinine is typically very low due to severe muscle wasting. In patients with renal impairment, serum urea is markedly elevated, whereas creatinine may rise above baseline but often remains within the reference range. Although creatinine-based glomerular filtration rate (eGFR) systematically overestimates renal function [20,21,22,23,24,25,26,27], it is still widely used in clinical practice due to the lack of validated alternatives for DMD.

Cystatin C (CysC), a cysteine protease inhibitor and established biomarker of kidney function, may represent a more reliable parameter in this patient population, as it is freely filtered by the glomerulus and not affected by muscle mass [28]. CysC therefore appears to be a suitable alternative for detecting early renal impairment in Duchenne and Becker muscular dystrophy, as well as in other neuromuscular diseases [27,28,29]. A significant correlation has been reported between CysC-based eGFR and left ventricular ejection fraction following the onset of systolic dysfunction, suggesting that CysC may also provide early evidence of cardiorenal syndrome in this population [30]. However, CysC levels may be modestly influenced by corticosteroid therapy, with reported changes not consistently associated with overt renal dysfunction [27,31].

Additional urinary biomarkers have been investigated in DMD (Figure 3). Proteomic and metabolomic analyses revealed possible biomarkers of muscle- and systemic-related changes, including titin (*TTN*) fragments [32], elevated levels of tetranor-PGDM, a major metabolite of prostaglandin D2 [33], and high urinary ferritin [34]. Elevated ferritin levels appear functionally linked to renal handling of myoglobin-derived iron released from dystrophic muscle, providing new insights into iron metabolism and kidney function in DMD [34].

Uromodulin, a protein produced exclusively in the thick ascending limb of Henle’s loop and distal tubule, has also been proposed as a kidney-specific biomarker and index of chronic renal damage [35,36]. Increased urinary uromodulin has recently been reported in DMD patients, consistent with underlying renal injury [37]. In addition, a prospective study of 52 DMD patients (April 2012–August 2023) evaluated longitudinal changes in blood urea nitrogen (BUN). Although BUN may be elevated in non-renal conditions, 30% of patients with BUN ≤ 30 mg/dL were considered to have renal dysfunction, and two patients died of renal failure ([38], abstract, WMS 2024, unpublished). Beyond classical plasma and urinary biomarkers, proteomic and metabolomic studies are beginning to uncover novel signatures that may reflect both muscle-derived factors and intrinsic kidney involvement in DMD. These omics-based biomarkers remain under investigation and require validation in larger patient cohorts [36].

Cardiomyopathy is a major feature of DMD and, alongside respiratory failure, represents a leading cause of morbidity and mortality [39]. With advances in respiratory and orthopedic care extending survival, the secondary effects of cardiomyopathy on other organs, including the kidney, are becoming increasingly evident. Consistent with this framework, cardiac and renal dysfunction in DMD can be interdependent, as in the general population [8,30].

In DMD, renal involvement has been linked to chronic cardiac dysfunction, inadequate fluid intake, and diuretic use [1,40,41,42]. Matsumura et al. (2012) described six adult DMD patients who developed acute renal failure, often in association with chronic fluid restriction, discontinuation of fluid support, excessive diuretic use, or diarrhea [41]. Importantly, DMD kidneys appear especially vulnerable to hypoperfusion, placing patients at high risk of acute or chronic renal failure during periods of fluid deprivation. Although vascular abnormalities and impaired blood flow are well documented in DMD muscle [43], no studies have yet demonstrated renal blood flow impairment in this population [8,40]. Case reports highlight further examples of renal vulnerability. Alhaswani et al. ([44] abstract, Neuromuscular Disorders, 2016) reported two male patients with DMD who developed marked renal deterioration, although this observation was preliminary and based on limited data [44]. One 18-year-old non-ambulant patient with severe cardiomyopathy developed acute kidney injury due to hypoperfusion and severe anemia, requiring repeated transfusions. A second 16-year-old patient experienced transient renal impairment following a chest infection and urinary tract infection.

Cardio–renal–anemia (CRA) syndrome, first described by Silverberg et al. (2006), describes a self-perpetuating cycle linking cardiac dysfunction, renal impairment, and anemia [45]. Anemia reduces oxygen delivery and increases cardiac workload, while impaired renal perfusion limits erythropoietin (EPO) production, exacerbating both anemia and heart failure. Although anemia is not a primary feature of DMD, it becomes increasingly common in patients with advanced cardiorenal involvement. In the case series by Alhaswani et al. (2016), anemia was a common finding in six patients who died of acute kidney injury [44]. Similarly, Motoki et al. (2015) reported four non-ambulatory patients with concurrent anemia and renal dysfunction, suggesting that reduced oxygen-carrying capacity may have increased cardiac workload and contributed to renal hypoperfusion [8]. Importantly, preserved renal function and correction of anemia are associated with improved outcomes in chronic heart failure [46], supporting their relevance in DMD, particularly as cardiomyopathy progresses. These observations underscore the need for careful monitoring of renal function and hemoglobin levels in DMD, with early nephrology referrals when anemia or renal decline is detected [6,44]. The pathophysiology of anemia in DMD is likely multifactorial. Reduced renal perfusion or tubular–interstitial injury may impair EPO synthesis. Chronic systemic inflammation, a hallmark of late-stage DMD, may suppress erythropoiesis and disrupt iron metabolism, leading to anemia of chronic disease [47].

Complement system activation has also been implicated in anemia associated with cardiorenal syndromes [48], potentially through hemolysis or bone marrow suppression. In addition, long-term corticosteroid therapy, while beneficial for muscle and pulmonary function, may cause gastrointestinal bleeding or bone marrow suppression, further compounding anemia risk [49,50]. Collectively, these factors indicate that anemia should be considered an integral component of multisystemic decline in advanced DMD rather than an isolated abnormality. Targeted management of its underlying causes may help slow progression and improve outcomes. These observations further highlight the close interplay between cardiac and renal dysfunction in DMD.

Beyond cardiorenal and anemia-related interactions, nephrolithiasis has also been described in long-term survivors with DMD. In a retrospective cohort study, 20.7% (6/29) of DMD patients developed kidney stones compared with only 1.5% (1/68) of immobile patients with other neurological conditions, suggesting that DMD itself may increase the risk of symptomatic nephrolithiasis [51]. This complication adds further morbidity, including pain, hydronephrosis, and the need for interventions such as nephrostomy, with a significant impact on prognosis, management, and quality of life. Other renal abnormalities observed in DMD include mild proteinuria, hyper- or hypocalciuria, and hyperphosphaturia, potentially due to long-term exposure to muscle-derived metabolites [52]. With advancing age, urological manifestations also become more prevalent in DMD [52]. However, little is known about the mechanisms underlying bladder smooth muscle dysfunction, urinary incontinence, and their full clinical impact in dystrophinopathies [53,54].

Taken together, the available evidence, although limited and heterogeneous and largely reflecting the scarcity of large, dedicated clinical studies, supports the clinical relevance of renal dysfunction in advanced stages of Duchenne muscular dystrophy and underscores the need to include routine renal monitoring as part of multidisciplinary patient care [30]. This perspective is consistent with adult heart failure guidelines [55] and with the National Institute for Health and Care Excellence (NICE) recommendations on chronic heart failure (CG108, 2010), which identify renal failure as an independent predictor of mortality and adverse cardiovascular outcomes [54,55,56].

### 2.3. Renal Impairment in Pediatric DMD Patients

Although renal dysfunction, often associated with worsening cardiac function, has been described in adult patients with DMD, kidney involvement in pediatric patients has received far less attention, despite the recognized susceptibility of the pediatric kidney to acute injury in the context of systemic diseases [57]. Kutluk and Doğan (2020) reported that serum cystatin C levels positively correlate with serum creatine kinase (CK) in children with DMD, suggesting that acute and progressive muscle breakdown during childhood may place increased metabolic stress on the kidneys [52,58]. However, longitudinal prospective studies are required to clarify the contribution of progressive muscle degeneration to the onset and progression of CKD in this population [52].

Supporting the limited data available in pediatric DMD cohorts, a prospective study demonstrated a high prevalence of hyperfiltration and hypertension in children and adolescents with DMD. Since most hypertensive patients were receiving corticosteroid therapy, an iatrogenic effect cannot be excluded. This finding underscores the need to investigate more thoroughly the long-term impact of chronic corticosteroid use on renal function in DMD [20].

A recent case report described a patient with steroid-dependent nephrotic syndrome (NS), histologically confirmed as minimal change NS, who subsequently developed a proximal muscle disorder and was later diagnosed with DMD. Genetic testing identified a deletion of exons 45–46 in the DMD gene. Previously, only one other case had been reported, involving a child with NS in whom biopsy revealed mesangial proliferative glomerulonephritis; this child was later diagnosed with DMD due to a deletion in exon 52 of the dystrophin gene. Clarifying whether Duchenne muscular dystrophy and nephrotic syndrome can coexist coincidentally or are mechanistically linked could have important implications for both diagnosis and clinical management [59]. In pediatric Duchenne muscular dystrophy, however, evidence on renal involvement remains particularly limited and is largely derived from small cohorts and isolated reports. The relative brevity of this section therefore reflects the current scarcity of dedicated pediatric data rather than a lower clinical relevance, consistent with broader challenges in generating longitudinal renal evidence in pediatric populations, and underscores the need for dedicated prospective studies in this population [60].

### 2.4. Drug-Induced Nephrotoxicity

In Duchenne muscular dystrophy, drug-induced nephrotoxicity should be considered within the broader context of disease-related multisystem involvement and long-term pharmacological management. Renal function is not only a potential target of drug toxicity but also a key determinant of drug exposure, therapeutic response, and safety, particularly in advanced disease stages characterized by cardiorenal vulnerability and extensive polypharmacy. Therapeutic strategies in DMD primarily aim to improve the structural integrity of muscle fibers and restore dystrophin expression or to mitigate downstream consequences of dystrophin deficiency, including inflammation, fibrosis, impaired regeneration, and muscle wasting [61]. In this context, long-term pharmacological treatments, such as corticosteroids, cardioprotective agents, and emerging exon-skipping drugs, are widely employed and have significantly improved clinical outcomes.

However, drug-induced nephrotoxicity remains a clinically relevant concern, particularly in DMD patients who are frequently exposed to long-term polypharmacy (Table 1), within a broader clinical framework in which drug-induced acute kidney injury is a well-recognized complication across multiple drug classes and clinical settings [62,63]. While nephrotoxicity is a well-recognized complication of several drug classes in the general population, its impact may be amplified in DMD due to underlying multisystem fragility, recurrent dehydration, impaired cardiac output, and exposure to nephrotoxic agents such as non-steroidal anti-inflammatory drugs (NSAIDs) and iodinated contrast media. Aminoglycosides, although classically nephrotoxic, may occasionally be administered for intercurrent infections or have been explored experimentally as readthrough therapy in patients with nonsense mutations; notably, no renal toxicity was observed in short-term gentamicin regimens reported in DMD [64,65], but caution remains warranted given the well-known class risk [66]. Indeed, cases of acute kidney injury in DMD patients undergoing polypharmacy have been reported [67], underscoring the need for careful renal monitoring in this vulnerable population.

With regard to glucocorticoids, the current standard of care in DMD [67,68,69], no overt renal toxicity has been reported. However, long-term corticosteroid use may contribute indirectly to renal strain through systemic effects such as hypertension and fluid retention, which warrant regular monitoring.

By contrast, caution is warranted with drugs prescribed for cardiac complications. As in the general population, heart failure in DMD is managed with β-blockers and renin–angiotensin system inhibitors, including angiotensin-converting enzyme (ACE) inhibitors and angiotensin receptor blockers (ARBs) [2]. In addition, newer cardioprotective drugs such as sodium–glucose cotransporter 2 (SGLT2) inhibitors and angiotensin receptor–neprilysin inhibitors have attracted interest as potential therapies in DMD, although their efficacy and safety in this population remain to be established [54].

However, it is important to emphasize that SGLT2 inhibitors are not part of the current standard of care in DMD. Their use in this setting remains purely experimental, supported only by mechanistic rationale derived from studies in diabetic and non-diabetic heart failure populations. Ongoing research will be essential to clarify their role, including the dedicated Phase IIa open-label trial currently evaluating empagliflozin for DMD-associated cardiomyopathy in children and adolescents (Repurposing Empagliflozin for DMD-associated Cardiomyopathy in Children & Adolescents 6–18 Years; ClinicalTrials.gov NCT06643442). These studies will help determine whether SGLT2 inhibitors can be safely and effectively integrated into future DMD management. In children with dilated cardiomyopathy, ACE inhibitors and/or β-blockers can also help prevent or delay disease progression [2,3,4].

The risk of drug-induced renal injury in DMD should be contextualized according to drug class. Certain widely used medications, such as ACE inhibitors and NSAIDs, can reduce renal perfusion by lowering glomerular filtration pressure, especially under conditions of volume depletion or low sodium intake. Prolonged exposure to these agents, particularly in patients with advanced cardiomyopathy and altered hemodynamics, may increase susceptibility to acute kidney injury [8,62]. By contrast, newer drug classes such as SGLT2 inhibitors (gliflozins) have demonstrated renoprotective effects in both diabetic and non-diabetic patients with heart failure, despite an initial reduction in estimated glomerular filtration rate (eGFR) due to decreased intraglomerular pressure [70,71].

Unlike NSAIDs or ACE inhibitors, SGLT2 inhibitors (gliflozins) attenuate intraglomerular hypertension while preserving long-term kidney function [70,71,72]. Given the increasing prevalence of polypharmacy in adult DMD patients, careful drug selection based on renal safety profiles is essential. A summary of the most relevant drug classes and their renal implications is provided in Table 1.

Beyond symptomatic management, disease-modifying therapies aimed at restoring dystrophin expression are also advancing. These include small molecules, antisense oligonucleotides (ASOs), and gene therapy approaches using adeno-associated virus (AAV) vectors [73,74,75,76,77,78,79]. Among these, nephrotoxicity is a potential concern with ASO therapies. While preclinical models and reviews of marketed ASO drugs demonstrate renal accumulation and possible renal injury, clinical evidence in DMD patients remains limited by small cohorts and short follow-up [80,81,82].

Currently, only a few ASOs, golodirsen, viltolarsen, and casimersen, have received FDA approval for DMD, whereas other agents such as nusinersen and inotersen target different diseases. Nevertheless, class-wide concerns about drug-induced kidney injury (DIKI) persist. Preclinical studies have repeatedly shown ASO-associated renal damage, including tubular degeneration, glomerulonephritis, and proteinuria, leading to FDA safety warnings for these drugs [81]. In contrast, long-term clinical data on golodirsen have so far indicated an overall favorable safety profile without clear evidence of nephrotoxicity, although continued monitoring remains warranted [83]. Although overt nephrotoxicity has not yet been demonstrated in limited human trials, long-term renal monitoring remains prudent in patients receiving ASOs. Mechanistically, kidney-associated toxicity from ASOs often results from renal accumulation of parent compounds and degradation products originating from other organs, such as the liver [84]. Long-term pharmacovigilance and renal monitoring should therefore be systematically incorporated into future DMD trials with ASOs and other innovative therapies. For several pharmacological classes currently used in the clinical management of Duchenne muscular dystrophy, renal safety data remain largely extrapolated from other clinical settings, underscoring the need for cautious interpretation and systematic renal monitoring in this population, in line with broader evidence on drug-induced nephrotoxicity [85].

### 2.5. Kidney Function in Animal Models of DMD

Given the renal abnormalities observed in patients with dystrophinopathy, the use of preclinical animal models is of particular interest for investigating proteome-wide changes in the kidney in relation to dystrophin deficiency. To date, only one systematic study has examined the renal proteome in a dystrophic mdx-4cv mouse model. Among 5878 renal proteins identified by mass spectrometry, 82 were decreased and 142 increased in association with muscular dystrophy [86].

Notably, this proteomic analysis revealed a marked elevation of the FABP1 isoform of fatty acid–binding protein [86,87], potentially reflecting ectopic lipid deposition and disrupted lipid metabolism. This finding positions FABP1 as an interesting biomarker candidate for assessing renal dysfunction in animal models of X-linked muscular dystrophy [86]. The mdx mouse, a well-established genetic model of DMD, has also been employed to study cellular abnormalities in the kidney [17] and to evaluate renal toxicity in pharmacological studies, including exon-skipping therapies [88]. In several small studies, no major histological abnormalities or changes in kidney weight were detected in mdx mice compared with wild-type controls, although available data are limited and do not exclude subtle or functional renal alterations [22,86].

However, subtle molecular and functional changes may occur and may not be evident on routine histological evaluation. For example, Gusel’nikova et al. (2018) reported the presence of amyloid deposits in the kidneys of aged mdx mice (18–20 months), pointing to a possible link between amyloid formation and DMD, and providing an opportunity to study the natural progression of amyloidosis in dystrophin-deficient tissues [17]. Supporting this, Wada et al. (2019) demonstrated that renal involvement contributes to the pathogenesis of mineral and bone disorders in mdx mice, highlighting the importance of considering both disease stage and systemic complications when evaluating renal pathology in DMD models [22].

Furthermore, more recent work suggests that renal dysfunction may contribute to disturbances in mineral and bone metabolism in muscular dystrophy. Specifically, mdx mice appear to be particularly vulnerable to dietary phosphorus overload, providing new insights into the interplay between renal, musculoskeletal, and metabolic health in dystrophinopathy [22]. Overall, despite limited evidence for consistent macroscopic renal abnormalities, the mdx mouse remains a valuable model for exploring renal pathophysiology and systemic complications associated with DMD, within a broader preclinical framework in which animal models have primarily been used to investigate skeletal and cardiac manifestations [89].

DMD, caused by dystrophin gene mutations, is characterized not only by progressive skeletal muscle wasting and fatty infiltration but also by systemic involvement of multiple organs. Clinical features include scoliosis and joint contractures, dilated cardiomyopathy, arrhythmias, and heart failure, progressive respiratory insufficiency, cognitive and behavioral disturbances, metabolic and endocrine abnormalities, gastrointestinal and hepatic dysfunction, and emerging evidence of renal and urinary tract involvement. This multisystemic presentation underscores the need for an integrative approach to patient care and research.

Dystrophin deficiency and membrane instability, chronic cardiac complications, and possible drug-induced nephrotoxicity represent factors that may contribute to renal involvement in DMD. Although the underlying mechanisms remain uncertain, several functional alterations have been reported in patients and animal models, including reduced renal perfusion, nephrolithiasis, mild proteinuria, mineral metabolism disturbances (hypercalciuria, hypocalciuria, hyperphosphaturia), and lower urinary tract dysfunction with incontinence. These findings highlight the kidney as an emerging target organ within the multisystemic pathophysiology of DMD.

Classical plasma biomarkers (e.g., creatinine, urea, cystatin C; red dashed arrow) and urinary biomarkers (e.g., titin fragments, ferritin, uromodulin; yellow dashed arrows) provide indirect evidence of renal impairment in DMD. In addition, intrinsic renal alterations (blue dashed arrow) may also contribute to disease-related signatures. Emerging omics-based approaches, including proteomic and metabolomic profiling, are uncovering novel biomarker candidates that integrate signals from both plasma- and urine-derived factors as well as from intrinsic kidney involvement.

This table summarizes the main drug classes employed in the management of DMD, including standard therapies and emerging agents. Reported renal implications are based on clinical and preclinical evidence, as detailed in the main text.

## 3. Integrative Discussion of Multisystem Involvement and Clinical Considerations

The evidence summarized in this review supports the view that renal involvement in Duchenne muscular dystrophy represents an emerging and clinically relevant component of multisystem disease progression rather than an isolated complication.

Recent clinical observations indicate that clinically significant renal injury may occur in patients with Duchenne muscular dystrophy even in the presence of apparently preserved serum creatinine levels, highlighting the risk of unrecognized kidney involvement [90]. Although direct structural kidney damage due to dystrophin deficiency remains poorly defined, multiple converging factors, including chronic cardiomyopathy, altered hemodynamics, systemic inflammation, anemia, and long-term pharmacological exposure, create a context of heightened renal vulnerability as survival improves.

From a clinical standpoint, this complexity challenges the reliability of conventional renal biomarkers and highlights the need for adapted monitoring strategies. The limitations of creatinine-based estimates in patients with severe muscle wasting underscore the value of alternative markers, such as cystatin C and selected urinary biomarkers, not as definitive diagnostic tools but as components of a longitudinal, integrative assessment.

Recent reviews of the biomarker landscape in Duchenne muscular dystrophy emphasize that no single biomarker adequately captures multisystem involvement and support the use of integrated, context-dependent approaches tailored to disease stage and comorbidities [36,91]. Importantly, renal function should be viewed as an active modifier of clinical trajectories in DMD, influencing drug handling, tolerance to polypharmacy, and vulnerability to acute decompensation during intercurrent illness or volume stress. Within this framework, kidney dysfunction may act as a silent amplifier of cardiorenal and cardiorenal–anemia interactions, contributing to systemic decline in advanced disease stages. Taken together, these considerations reinforce the need to systematically incorporate renal assessment into multidisciplinary DMD care pathways and clinical trials, not only for safety monitoring but also for risk stratification and individualized therapeutic decision-making.

## 4. Conclusions

With the progressive improvement of standards of care, life expectancy in patients with DMD has increased substantially. As a result, renal dysfunction is increasingly recognized as an important comorbidity within the multisystemic pathophysiology of DMD. However, current evidence on renal involvement in Duchenne muscular dystrophy remains limited and heterogeneous, reducing our understanding of its true incidence, histopathological features, reliable biomarkers, and underlying mechanisms. The data reviewed here underscore the need for systematic preclinical and clinical studies to better characterize renal involvement in DMD and highlight the importance of incorporating routine renal monitoring into the multidisciplinary management of these patients. In addition, potential renal safety issues should be carefully considered during the clinical development and long-term use of pharmacological therapies for DMD. Overall, renal alterations, though often underrecognized, may actively contribute to systemic complications and disease progression. A clearer understanding of kidney involvement may therefore represent an important opportunity to improve risk stratification and long-term outcomes in patients with dystrophinopathy.

## Figures and Tables

**Table 1:** Pharmacological treatments used in DMD and renal implications.

Drug Class	Use in DMD	Renal Implications
*Corticosteroids* (e.g., *prednisone*, *deflazacort*)	Standard of care; reduce inflammation, delay progression.	No clinically evident nephrotoxicity reported to date.
*β-blockers*	Management of cardiomyopathy/heart failure.	No evidence of direct nephrotoxicity; potential effects under reduced renal perfusion.
*ACE inhibitors*/*ARBs*	First-line therapy for cardiomyopathy.	Risk of reduced renal perfusion and acute kidney injury, particularly under conditions of dehydration or low sodium intake.
*NSAIDs*	Symptomatic use (e.g., pain, fever).	Nephrotoxicity well documented; risk of reduced perfusion and AKI.
*SGLT2 inhibitors* (*gliflozins*)	Investigational cardioprotective therapy.	Demonstrated renoprotective effects in the general population; transient eGFR decline followed by long-term preservation.
*ARNI* (*sacubitril*/*valsartan*)	Potential therapy for cardiomyopathy.	Safety and renal effects have not yet been established in DMD patients.
*ASOs* (e.g., *golodirsen*, *viltolarsen*, *casimersen*)	Exon skipping to restore dystrophin.	Preclinical studies show renal accumulation and toxicity, while clinical data remain limited; renal monitoring is recommended.
*AAV-based gene therapies*	Experimental, aiming to restore dystrophin.	Limited data; renal safety under investigation.

## Data Availability

No new data were created or analyzed in this study.

## Abbreviations

The following abbreviations are used in this manuscript:

DMD	Duchenne muscular dystrophy
